# PXL01 in sodium hyaluronate results in increased PRG4 expression: a potential mechanism for anti-adhesion

**DOI:** 10.1080/03009734.2016.1230157

**Published:** 2016-09-23

**Authors:** Sara Edsfeldt, Björn Holm, Margit Mahlapuu, Carol Reno, David A. Hart, Monica Wiig

**Affiliations:** a Department of Surgical Sciences, Hand Surgery, Uppsala University, Uppsala, Sweden;; b Department of Hand Surgery, Uppsala University Hospital, Uppsala, Sweden;; c Pergamum AB, Stockholm, Sweden;; d Department of Surgery, McCaig Institute for Bone and Joint Health, University of Calgary, Calgary, Canada

**Keywords:** Adhesions, flexor tendons, lubricin, proteoglycan 4, PXL01

## Abstract

**Purpose:**

To investigate the anti-adhesive mechanisms of PXL01 in sodium hyaluronate (HA) by using the rabbit lactoferrin peptide, rabPXL01 in HA, in a rabbit model of healing tendons and tendon sheaths. The mechanism of action for PXL01 in HA is interesting since a recent clinical study of the human lactoferrin peptide PXL01 in HA administered around repaired tendons in the hand showed improved digit mobility.

**Materials and methods:**

On days 1, 3, and 6 after tendon injury and surgical repair, reverse transcriptase-quantitative polymerase chain reaction (RT-qPCR) was used to assess mRNA expression levels for genes encoding the mucinous glycoprotein PRG4 (also called lubricin) and a subset of matrix proteins, cytokines, and growth factors involved in flexor tendon repair. RabPXL01 in HA was administered locally around the repaired tendons, and mRNA expression was compared with untreated repaired tendons and tendon sheaths.

**Results:**

We observed, at all time points, increased expression of *PRG4* mRNA in tendons treated with rabPXL01 in HA, but not in tendon sheaths. In addition, treatment with rabPXL01 in HA led to repression of the mRNA levels for the pro-inflammatory mediators interleukin (IL)-1β, IL-6, and IL-8 in tendon sheaths.

**Conclusions:**

RabPXL01 in HA increased lubricin mRNA production while diminishing mRNA levels of inflammatory mediators, which in turn reduced the gliding resistance and inhibited the adhesion formation after flexor tendon repair.

## Introduction

PXL01 is a synthetic peptide derived from the human milk protein, lactoferrin. *In vitro* studies in human cell lines have shown that PXL01 exhibits an inhibitory effect on important hallmarks of adhesion formation by reducing secretion of inflammatory cytokines, promoting fibrinolysis, and reducing infections (1). Further, PXL01 in sodium hyaluronate (HA) reduced post-surgical adhesions in experimental models of abdominal surgery in rats (1) and flexor tendon repair surgery in rabbits (2,3). A recent clinical trial investigating the efficacy and safety of adjuvant treatment with PXL01 in HA after flexor tendon injury and repair in the hand showed increased digit mobility in PXL01 in HA-treated fingers compared to placebo (4). However, the molecular mechanisms underlying the effect of PXL01 in HA on adhesion formation and tendon healing have not been clarified. In this study, we used the corresponding peptide derived from rabbit lactoferrin (rabPXL01) to study the effects in a rabbit model of tendon surgery. We hypothesized that treatment with rabPXL01 in HA would lead to reduced inflammation and increased synthesis of proteoglycan 4 (PRG4), also called lubricin, after tendon injury and repair in rabbits. Increased lubricin levels and reduced inflammation could subsequently lead to inhibition of adhesion formation.

*PRG4* is an interesting gene, and individuals carrying mutant variants of the gene can suffer a number of connective tissue complications (5). The protein it encodes is a mucinous glycoprotein secreted into synovial fluid by synovial fibroblasts (6) and the superficial zone cells of articular cartilage (7) and menisci (8). It has lubricating properties on articular cartilage (9), reduces synovial cell overgrowth, and protects the surface of the cartilage (10). Lubricin has also been found on the flexor tendon surface (11), and recent studies have shown that lubricin reduces tendon-gliding resistance (12,13). In the eye, the ocular surface epithelia express PRG4, which reduces friction between the cornea and conjunctiva (14). There is evidence that PRG4 may play a role in the biomechanics of rabbit ligaments during aging (15). PRG4 may also have anti-inflammatory effects itself (16). The expression of *PRG4* is inhibited by tumor necrosis factor α (TNF-α) and interleukin (IL)-1, and stimulated by transforming growth factor β (TGF-β) (11). Thus, interfering with these inflammatory molecules may potentially increase lubricin levels after a tendon injury.

To investigate if the potential anti-adhesive effect of rabPXL01 in HA is mediated via *PRG4*, directly or indirectly by inflammatory mediators, and to further characterize the molecular effects of rabPXL01 in HA on tendon healing, we assessed mRNA levels for PRG4 and a subset of relevant molecules involved in the outcome early after rabbit flexor tendon repair (Table 1). The aim of this study is to provide potentially important information regarding the possible early molecular basis for the effects of rabPXL01 in HA on healing tendons and tendon sheaths.

**Table 1.** Study data.

Molecules studied	Clinical relevance
Proteoglycan 4 (PRG4)/lubricin	Lubrication factor
Collagen I, collagen III	Matrix molecules
Cyclo-oxygenase 2 (Cox-2)	Inflammatory mediator
Inducible nitric oxide synthase (iNOS)	Inflammatory mediator
Interleukin 1β, 6, 8 (IL-1β, IL-6, IL-8)	Inflammatory mediator
Tumor necrosis factor α (TNF-α)	Inflammatory mediator
Transforming growth factor β (TGF-β)	Critical growth factor
α Smooth muscle actin (αSMA)	Myofibroblast marker

## Materials and methods

### Surgical procedure

Twenty-four female young adult New Zealand White rabbits weighing 3 kg (±0.3 kg) were divided into three groups (*n* = 8 for each group; killed 1, 3, or 6 days postoperatively). They were housed and cared for in accordance with regulations for the protection of laboratory animals. The local animal ethics committee approved the study (Permit Number: C 121/11). Rabbits were selected as experimental model since tendon repair surgery is feasible due to the sufficiently large size of the digit, while this type of surgery cannot be performed in mice or rats otherwise frequently used in non-clinical efficacy studies.

The surgical procedures were performed during anesthesia with fentanyl-fluanisone (0.3 mL/kg body weight; Hypnorm, Janssen, Beerse, Belgium) and midazolam (2 mg/kg body weight; Dormicum, Roche, Basel, Switzerland) under sterile conditions as described previously (17,18), and all efforts were made to minimize suffering. A prophylactic intravenous dose of the antibiotic cefuroxime (100 mg; Zinacef, GlaxoSmithKline, London, United Kingdom) was given before surgery. Buprenorfin (0.1 mg/kg body weight; Temgesic, RB Pharmaceuticals, Slough, United Kingdom) was given postoperatively to prevent pain. The surgical procedures were performed on both hind paws identically, as described previously (17,18). Briefly, to decrease the tensile load on the flexor tendon’s phalangeal section, a partial division of the tendons was performed at the tendon–muscle transition. The third digit was longitudinally incised, and the flexor tendon sheath opened approximately 10 mm between the first and second annular pulley. The superficial flexor tendon was resected, and the intermediate segment of the deep flexor tendon sharply divided transversely. The injured tendons were sutured end-to-end using a modified Kessler suture technique (5-0 Prolene, Ethicon, Sollentuna, Sweden) for the core suture, and then peripheral circumferential running sutures (6-0, PDS, Ethicon).

### Administration of rabPXL01 in HA

RabPXL01 was prepared in HA as previously described (2), using a concentration of 20 mg/mL rabPXL01 in 1.5% HA (molecular weight 1.5–8.1 × 10^6^ Da) (3). After surgery, the paws of the rabbits were randomized either to receive rabPXL01 in HA treatment or to be left untreated (paired control to account for any possible genetic influences on healing). RabPXL01 in HA was administered to the paw randomized to receive treatment (treatment paws were randomized for each animal and blinded to the surgeon up to the moment of product administration, and to all personnel involved in the evaluation) using a BD Neoflon 24GA catheter (Becton, Dickinson Infusion Therapy AB, Helsingborg, Sweden) connected to a 1 mL syringe containing the rabPXL01 in HA formula. The Neoflon catheter was inserted into the opening of the sheath and was still within the tendon sheath as the tendon sheath was closed with a running 6-0 PDS suture (Ethicon). When performing the final sutures, 0.3 mL of the rabPXL01 in HA formula was injected and the tendon sheath fully closed. The same procedure was performed in the untreated paw, but without injecting rabPXL01 in HA formula. The skin was closed with a running suture (5-0 Ethilon, Ethicon).

### Sample collection

The tendons and sheaths were harvested 1, 3, or 6 days after surgery. The rabbits were sedated with midazolam (Dormicum, Roche) and euthanized by a lethal dose of Pentobarbitalnatrium (Apoteket, Uppsala, Sweden). Equally sized segments of tendons and sheaths, 4 mm proximal and 4 mm distal of the repair site, were harvested. The samples were cleaned from sutures and rinsed in physiological saline. The samples were put into tared cryovials, immediately placed in liquid nitrogen, and stored at –80 °C until further analysis.

### RNA extraction and reverse transcriptase-quantitative polymerase chain reaction (RT-qPCR)

RNA was extracted from the tissue samples using the TRIspin method (19). The wet weight of the samples was assessed, and the frozen samples were pulverized using a Braun Micro-dismembrator (B. Braun Biotech International, Melsungen, Germany). After powdering, 1 mL of Trizol (Gibco Life Technologies, Carlsbad, CA) was added to each sample and the Trizol-powdered tissue was transferred to tubes and immediately put in liquid nitrogen until further analysis. The Trizol tubes were thawed at room temperature for subsequent analysis. After adding chloroform (0.3 mL; EMD Chemicals Inc., Gibbstown, NJ), the samples were centrifuged and the aqueous phase collected, to which ethanol was added to precipitate the RNA (70%, 0.6 mL). Using an RNeasy Total RNA Kit (Qiagen, Chatsworth, CA), RNA was eluted and quantified fluorometrically with Sybergreen (Mandel, Guelph, Ontario, Canada). RNA was further processed for reverse transcription to cDNA using a Qiagen Omniscript kit (Omniscript RT Kit, Qiagen, Mississauga, Ontario, Canada). cDNA was prepared for qPCR using IQ SYBR Green Supermix (Bio-Rad Laboratories Mississauga, Ontario, Canada). Primers previously utilized for rabbit tissues were added (Table 2) and the samples amplified using real-time PCR (iCycler iQ, Real Time PCR Detection System, Bio-Rad Laboratories). *18S* was used as the house-keeping gene for normalization, and non-reverse transcribed RNA was used as a negative control to detect possible DNA contamination (none of the samples contained detectable DNA; data not shown).

**Table 2.** Primer sequences.

Gene	Primer sequence		Base pairs	Source
18S	Forward sequence	TGG TCG CTC GCT CCT CTC C	360	NR_003286
	Reverse sequence	CGC CTG CTG CCT TCC TTG G		
αSMA	Forward sequence	GTG TGA GGA AGA GGA CAG CA	446	X60732
	Reverse sequence	TAC GTC CAG AGG CAT AGA GG		
Collagen I	Forward sequence	GAT GCG TTC CAG TTC GAG TA	312	Personal communication
	Reverse sequence	GGT CTT CCG GTG GTC TTG TA		
Collagen III	Forward sequence	TTA TAA ACC AAC CTC TTC CT	255	Personal communication
	Reverse sequence	TAT TAT AGC ACC ATT GAG AC		
COX-2	Forward sequence	CAA ACT GCT CCT GAA ACC CAC TC	82	NM_001082388
	Reverse sequence	GCT ATT GAC GAT GTT CCA GAC TCC		
IL-1β	Forward sequence	GCC GAT GGT CCC AAT TAC AT	121	M26295
	Reverse sequence	ACA AGA CCT GCC GGA AGC T		
IL-6	Forward sequence	CCT GCC TGC TGA GAA TCA CTT	51	AF469048
	Reverse sequence	CGA GAT ACA TCC GGA ACT CCA T		
IL-8	Forward sequence	CAA CCT TCC TGC TGT CTC TG	145	NM_001082293
	Reverse sequence	GGT CCA CTC TCA ATC ACT CT		
iNOS	Forward sequence	CTG TGA CGT CCA GCG CTA CA	119	AF469048
	Reverse sequence	GCA CGG CGA TGT TGA TCT CTC GCC CT		
PRG4	Forward sequence	GAA CGT GCT ATA GGA CCT TC	287	NM_00127709
	Reverse sequence	CAG ACT TTG GAT AAG GTC TGC C		
TGF-β	Forward sequence	CGG CAG CTG TAC ATT GAC TT	271	AF000133
	Reverse sequence	AGC GCA CGA TCA TGT TGG AC		
TNF-α	Forward sequence	TCT AGT CAA CCC TGT GGC CC	51	NM_00108
	Reverse sequence	GCC CGA GAA GCT GAT CTG AG		

### Statistical analysis

Levels of mRNA for a subset of mediators involved in the healing of flexor tendons and tendon sheaths were assessed 1, 3, and 6 days postoperatively. Flexor tendons and tendon sheaths treated with rabPXL01 in HA were compared with its untreated paired control using repeated measures ANOVA. Table 3 is a supplement reporting the remaining data. All tests were two-sided, and *P* < .05 was considered statistically significant.

**Table 3.** Influence of rabPXL01 in HA on mRNA expression of matrix proteins, growth factors, and inflammatory mediators by injured tendons and tendon sheaths.

		Percent of corresponding control (untreated tendons and tendon sheaths)			*P* values		
Molecule	Group	Day 1	Day 3	Day 6	Day 1	Day 3	Day 6
Collagen I	Tendon PXL01	390.2	144.2	112.3	.13	.387	.742
	Sheath PXL01	60.1	87.2	42.2	.494	.563	.082
Collagen III	Tendon PXL01	973.9	157.8	96.4	.003	.387	.889
	Sheath PXL01	70.8	69.9	58.4	.556	.563	.065
Cox-2	Tendon PXL01	263.5	121.1	93.8	.214	.506	.896
	Sheath PXL01	71.7	76	56.7	.331	.416	.094
iNOS	Tendon PXL01	398	175.7	232.6	.085	.043	.298
	Sheath PXL01	114.7	98.9	55.1	.687	.969	.106
IL-1β	Tendon PXL01	153.6	110.3	82.7	.194	.782	.581
	Sheath PXL01	64.6	95.7	51.7	.432	.876	.04
IL-6	Tendon PXL01	177.6	154.4	163.7	.337	.233	.582
	Sheath PXL01	82.2	81.5	49	.789	.607	.005
IL-8	Tendon PXL01	192.9	110.7	88.4	.093	.79	.744
	Sheath PXL01	39.8	106	53.6	.265	.824	.009
TNF-α	Tendon PXL01	89.9	131.9	126.4	.742	.473	.441
	Sheath PXL01	66.9	84.9	56.8	.427	.553	.129
TGF-β	Tendon PXL01	189.8	112	92	.256	.724	.554
	Sheath PXL01	45.4	74	48.6	.409	.304	.106
αSMA	Tendon PXL01	333	133.2	154.4	.367	.602	.447
	Sheath PXL01	43.1	63.2	56.9	.304	.161	.139

Levels of mRNA in tendons and tendon sheaths for each molecule are shown as percent of corresponding control (the tendon and tendon sheath of the untreated paw). *P* values are shown for each molecule on days 1, 3, and 6 after surgery.

## Results

### Rupture rate

Three tendon ruptures occurred after repair surgery (two ruptures in the same rabbit): two of the untreated tendons and one of the rabPXL01 in HA-treated tendons, giving a rupture rate of 8% for the untreated tendons and 4% for the rabPXL01 in HA-treated tendons.

### The effect of rabPXL01 in HA on the expression of PRG4 mRNA in tendon and tendon sheaths

**Figure 1.**
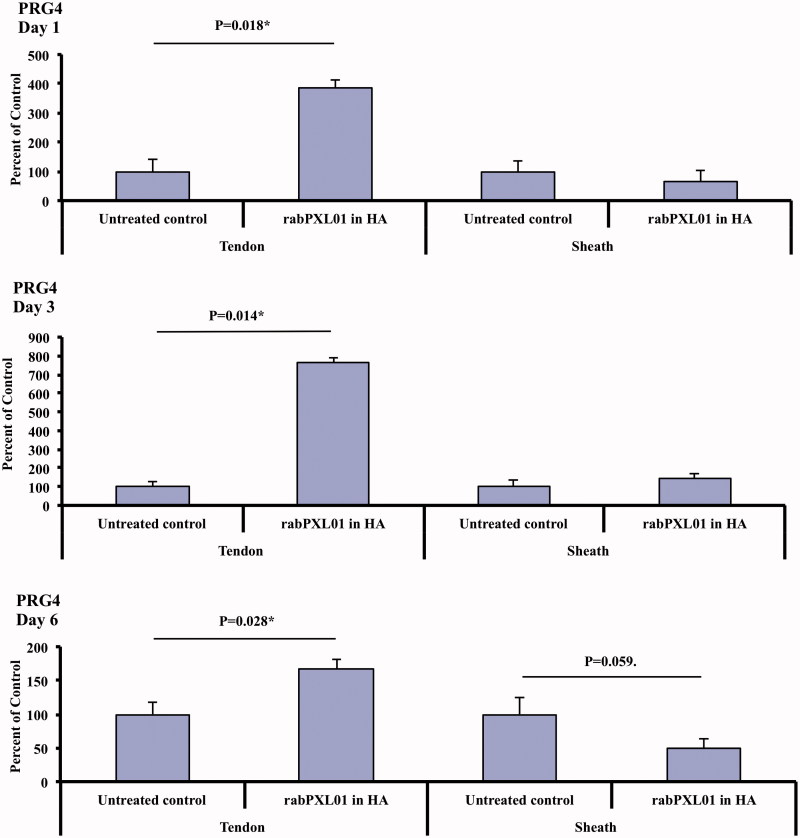
Expression of *PRG4* mRNA in flexor tendons and tendon sheaths treated with rabPXL01 in HA compared to untreated tendons and tendon sheaths 1, 3, and 6 days postoperatively. The following significance symbols are used: ***P < .001; **P < .01; *P < .05.

At all time points levels of *PRG4* mRNA were higher in tendons treated with rabPXL01 in HA compared to untreated tendons. On the other hand, levels of *PRG4* mRNA were similar in treated and untreated sheaths (Figure 1).

### Influence of rabPXL01 in HA on mRNA levels for inflammatory mediators, transforming growth factor, the myofibroblast marker αSMA, and collagens I and III

**Figure 2.**
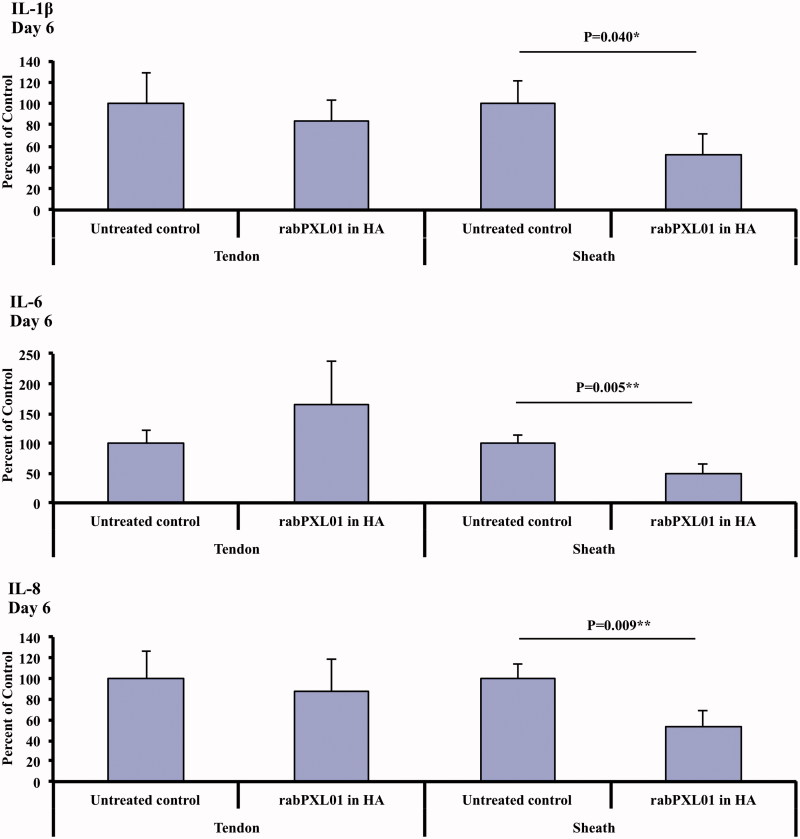
Expression of mRNA for IL-1β (top), IL-6 (middle), and IL-8 (bottom) in flexor tendons and tendon sheaths treated with rabPXL01 in HA compared to untreated tendons and tendon sheaths 6 days after surgery. The following significance symbols are used: ***P < .001; **P < .01; *P < .05.

In tendon sheaths treated with rabPXL01 in HA, mRNA levels of IL-1β, IL-6, and IL-8 were lower than in untreated sheaths on day 6 (Figure 2, Table 3). Levels of mRNA for iNOS tended to be higher in rabPXL01 in HA-treated tendons than in untreated tendons at all time points, but with significant differences detected only on day 3 (*P* = .043, Table 3). The mRNA level of collagen III in rabPXL01 in HA-treated tendons was higher than that of untreated tendons on day 1 (*P* = .003). RabPXL01 in HA treatment did not significantly influence mRNA levels for any of the other molecules assessed, neither in tendons nor in tendon sheaths (Table 3).

## Discussion

Previous studies on adjuvant treatment with PXL01 in HA have shown an increase in digit range of motion in both humans (4) and rabbits (2,3) without elevating the rate of rupture. The principal finding of the present study was an increased expression of *PRG4* mRNA on days 1, 3, and 6 after surgery in tendons treated with rabPXL01 in HA. These findings suggest that rabPXL01 in HA treatment leads to an increase in lubricin production, and subsequently reduces the gliding resistance and inhibits adhesion formations after flexor tendon repair. The present findings are consistent with those reported by Hayashi et al. (12) who compared tendons in *PRG4* knockout, heterozygous, and wild-type mice and showed that the gliding resistance of intrasynovial tendons from *PRG4* knockout mice was higher than in the other groups. Adjuvant treatment with lubricin has also been evaluated in canine models of flexor tendon repair (13,20–22). Carbodiimide-derivatized HA and lubricin-treated tendons exhibited lower gliding resistance than tendons treated with HA alone (13). However, two of these studies that showed positive effects on digit function also reported adverse effects on tendon healing and decreased repair strength (21,22). These investigations suggested that carbodiimide-derivatized HA and lubricin may prevent cellular adhesions to tendon surfaces and enable lubrication of the tendon during flexion and extension, but this treatment also interferes negatively with tendon healing. In the present study we observed higher levels of *PRG4* mRNA in rabPXL01 in HA-treated tendons, but no macroscopically obvious damage to the tendon tissue or altered rate of post-surgical tendon rupture. Previous studies using PXL01 in HA have shown no increase in the rate of tendon rupture and no tendon damages (2,3).

There were no detectable differences in mRNA levels in the tendons for the mediators IL-1, TNF-α, and TGF-β between treated and untreated groups. However, we found increased levels of *PRG4* mRNA in rabPXL01 in HA-treated tendons but not in tendon sheaths. Receptors for PXL01 are not presently known, but likely the differences between *PRG4* mRNA expression in tendons and tendon sheaths depend on PXL01 affinity for a tendon cell-specific receptor. Whether these cells are endogenous tendon cells or cells recruited to the injury site requires further investigation. These findings regarding *PRG4* point to an interesting new target on which to focus future investigations, as well as to confirm that the mRNA changes observed are followed by increased protein production and secretion.

We have previously shown that PXL01 has anti-inflammatory effects on human macrophages by reducing the release of pro-inflammatory cytokines IL-1β, IL-6, and IL-8 (1). Consistent with these observations, the present study revealed a significant and co-ordinated suppression of mRNA levels for IL-1β, L-6, and IL-8 in tendon sheaths 6 days after surgery. Prolonged inflammation in the wound-healing cascade can lead to excessive adhesion formation, whereas down-regulation of the inflammatory response is believed to restrict proliferation and remodeling, leading to prevention of scarring (23). Suppression of inflammation may thus be one additional complimentary mechanism behind the improvement in digit mobility that has been shown after adjuvant treatment with PXL01 in HA (2–4).

The healing mechanisms in the tendon differ from the mechanisms operative in the tendon sheath. There is, for example, a change in collagen expression with increasing type III collagen in both the injured tendon and tendon sheath directly after an injury, whereas the level of collagen I remains unaltered in the tendon and increases in the tendon sheath at a later stage (17). This shift in collagen expression is believed to decrease tendon strength (24). In the present study, we observed higher levels of mRNA for both collagen I and collagen III in tendons treated with rabPXL01 in HA, at the earliest time point studied, day 1. An increase of both collagen I and collagen III production early in the healing process might create a stronger tendon tissue that can better withstand forces created during early active rehabilitation, with limited risk of repair rupture (24). Therefore, increased production of collagens I and III early after rabPXL01 in HA treatment might help to reduce the risk of repair rupture, but further studies are needed to support this notion.

HA as carrier allows for a slow release of rabPXL01 combined with initial lubricating properties around the tendon. HA has also shown some anti-inflammatory effects itself by reducing the concentration of inflammatory mediators (25). The current study design does not enable us to differentiate between the molecular effects of rabPXL01 versus HA, which is a limitation of the experiment. However, there were several reasons for the chosen design. Firstly, this study design mimics the design of the recently published randomized clinical trial where the treatment with a combination of PXL01 in HA improved hand function (4). Secondly, in the same rabbit model of tendon injury, adjuvant treatment with the combination of PXL01 in HA resulted in improved digit mobility compared to HA alone (3). Finally, using PXL01 alone is not technically feasible in this *in vivo* model, as the peptide does not remain at the wound site when applied in saline, but rather diffuses away rapidly.

In this study, tissue samples were harvested 1, 3, and 6 days after surgery. These time points were selected to address potential changes in the expression profile of different wound-healing mediators. It is known that molecular events such as those associated with an inflammatory response, which subsequently ‘set the stage’ for scar formation, take place shortly after the injury (26). Relevant to this point, Berglund et al. (18) detected temporal alterations in the pattern of mRNA expression for IL-1β and hyaluronan synthases, which reached peak levels 3 and 6 days after tendon repair, respectively. A limitation of the present study is that mRNA expression may not completely mirror the protein levels, since there may be post-transcriptional regulation of protein synthesis.

In summary, our results suggest that the anti-adhesive effect of PXL01 in HA involves an increased production of PRG4 in tendons and a decreased expression of inflammatory mediators in tendon sheaths.
